# Evaluating the tumor immune profile based on a three-gene prognostic risk model in HER2 positive breast cancer

**DOI:** 10.1038/s41598-022-13499-1

**Published:** 2022-06-03

**Authors:** Jianqing Lin, Aiyue Zhao, Deqiang Fu

**Affiliations:** 1 Thyroid & Breast Surgery, The Second Affiliated Hospital of Fujian Medical University, Quanzhou, China; 2 Department of Oncology, The Second Affiliated Hospital of Fujian Medical University, Quanzhou, China

**Keywords:** Cancer, Computational biology and bioinformatics

## Abstract

To date, there have not been great breakthroughs in immunotherapy for HER2 positive breast cancer (HPBC). This study aimed to build a risk model that might contribute to predicting prognosis and discriminating the immune landscape in patients with HPBC. We analyzed the tumor immune profile of HPBC patients from the TCGA using the ESTIMATE algorithm. Thirty survival-related differentially expressed genes were selected according to the ImmuneScore and StromalScore. A prognostic risk model consisting of PTGDR, PNOC and CCL23 was established by LASSO analysis, and all patients were classified into the high- and low-risk score groups according to the risk scores. Subsequently, the risk model was proven to be efficient and reliable. Immune related pathways were the dominantly enriched category. ssGSEA showed stronger immune infiltration in the low-risk score group, including the infiltration of TILs, CD8 T cells, NK cells, DCs, and so on. Moreover, we found that the expression of immune checkpoint genes, including PD-L1, CTLA-4, TIGIT, TIM-3 and LAG-3, was significantly upregulated in the low-risk score group. All the results were validated with corresponding data from the GEO database. In summary, our investigation indicated that the risk model composed of PTGDR, PNOC and CCL23 has potential to predict prognosis and evaluate the tumor immune microenvironment in HPBC patients. More importantly, HPBC patients with a low-risk scores are likely to benefit from immune treatment.

## Introduction

Breast cancer is one of the most common carcinomas worldwide. Approximately 2.1 million new patients were reportedly diagnosed with breast cancer in 2018, and new patients with breast cancer accounted for approximately 24.2% of all cancer cases among women^1^. HPBC, a subtype of breast cancer, is characterized by the overexpression of HER2 protein^2^, shows drug resistance and highly aggressive biology, and is associated with poor survival^3–5^. Published data show that HPBC accounts for one-fourth to one-fifth of breast cancers^6^.

According to NCCN guidelines, the combination regimen of chemotherapy plus anti-HER2 drugs is regarded as the standard treatment regimen for HPBC patients. Although patient survival has been markedly prolonged by treatment with anti-HER2 drugs^7^, HPBC patients still face lack of optimal subsequent treatment after the development of drug resistance or tumor recurrence. In recent years, tumor immunotherapy, represented by PD-1/PD-L1 pathway blocking antibodies, has shown great success in various malignancies^8–12^, including triple-negative breast cancer^13^. Unfortunately, to date, there has been no great improvement in tumor immunotherapy for HPBC, except for a few reports in animal experiments^14^.

However, in the past few years, accumulating evidence has demonstrated a positive association between the tumor immune microenvironment (TIME) and the prognosis of HPBC patients. An increased number of stromal tumor-infiltrating lymphocytes (TILs) denotes a good prognosis in HPBC patients who received chemotherapy^15^. Coincidentally, high PD-L1 expression and marked CD8 TIL infiltration were regarded as markers of better outcome in HPBC patients treated with conventional chemotherapy and HER2-blocking therapy^16^. Recently, assessment of the TIME based on gene expression has attracted increasing attention in the HPBC field^17,18^. However, a recent phase II clinical trial showed that combination treatment with anti-Her2 and anti-PD-L1 agents did not improve progression-free survival (PFS) in HPBC patients^19^. Hence, it is crucial to accurately evaluate the TIME in HPBC patients.

In this study, we attempted to construct a risk model for predicting prognosis and evaluating the tumor immune profile in HPBC patients. Initially, we explored the immune-infiltrating profile of HPBC patients based on the ESTIMATE algorithm using data from the TCGA and GEO databases. Then, 30 survival-related genes were selected from 606 differentially expressed genes that were previously identified according to the ESTIMATE score. Finally, we created a gene-based risk model that may be helpful for accurately predicting prognosis and assessing the tumor-infiltrating immune profile in patients with HPBC. With this model, we hope to provide personalized tumor immunotherapy for appropriate patients with HPBC in the future.

## Results

### Characteristics of the cohorts

A total of 280 HER2-positive patients from the TCGA, GSE20711, GSE45255, GSE162228 and GSE1456 databases were selected for our study. The training cohort contained 132 patients from the TCGA. The validation cohort comprised 26 patients from GSE20711, 61 from GSE45255, 46 from GSE162228 and 15 from GSE1456. The accuracy of the constructed model was verified by a validation cohort. The clinical characteristics of the HPBC patients are shown in Table S1.

### Immune infiltration is a survival-associated factor in HPBC patients

**Figure 1:**
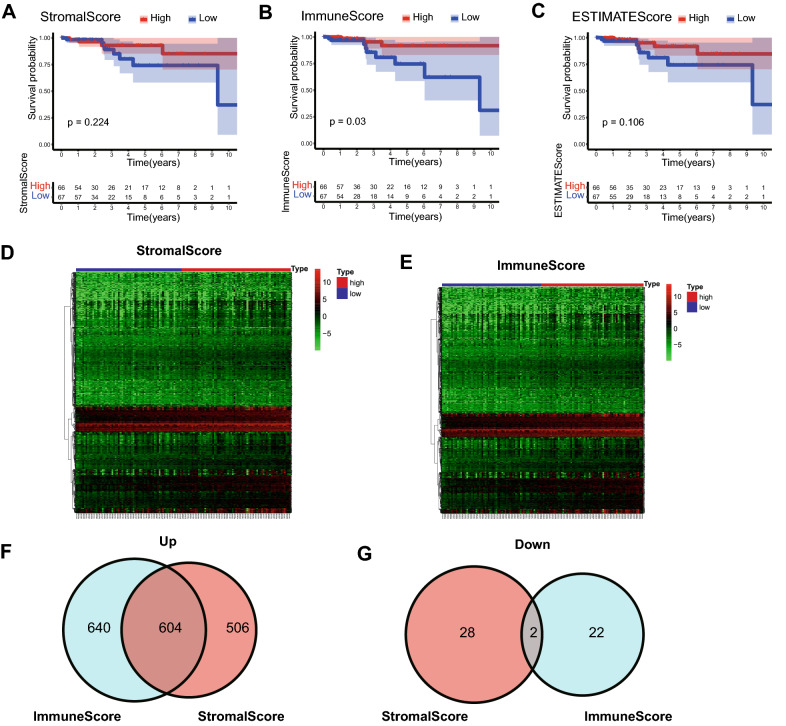
Survival analysis and differentially expressed genes based on the ESTIMATE algorithm in the training cohort. (**A**–**C**) Kaplan–Meier survival curves of patients grouped by the median of StromalScore, ImmuneScore and ESTIMATEScore. (**D**,**E**) Heatmap of differentially expressed genes between the high score group and the low score group from StromalScore data and ImmuneScore data. (**F**) Venn diagram of the upregulated differentially expressed genes from StromalScore data and ImmuneScore data. (**G**) Venn diagram of the downregulated differentially expressed genes from StromalScore data and ImmuneScore data.

To evaluate immune cell infiltration in HPBC patients, the ESTIMATE algorithm was utilized to calculate the ESTIMATE scores for the TCGA cohort, reflecting the TIME landscape^20^. The median scores, including the StromalScore, ImmuneScore and ESTIMATEScore, were assigned as the cutoff values. All patients were classified into the corresponding low- and high-risk groups. The results of the Kaplan–Meier survival analysis showed that the overall survival (OS) in the high ImmuneScore group was better than that in the low ImmuneScore group (*p* = 0.03), but no survival difference was found between the groups based on the StromalScore (*p* = 0.224) or the ESTIMATEScore (*p* = 0.106) (Fig. 1A–C). We further analyzed the correlations of OS with ImmuneScore, age and stage by univariate and multivariate Cox analyses. Unexpectedly, the results showed that the ImmuneScore could not be regarded as an independent prognosis-related factor in the TCGA cohort (Fig. S1).

Subsequently, we explored differential gene expression between groups based on the StromalScore and ImmuneScore. In the StromalScore groups, 1140 differentially expressed genes (DEGs) were identified (|log FC|≥ 1, *p* < 0.05). Among them, 1110 genes were significantly upregulated and 30 genes were significantly downregulated (Fig. 1D). Similarly, among 1268 DEGs identified in the ImmuneScore groups, 1244 were significantly upregulated and 24 were significantly downregulated (|log FC|≥ 1, *p* < 0.05) (Fig. 1E). Furthermore, 606 DEGs (604 upregulated genes and 2 downregulated genes) were identified in both the StromalScore groups and ImmuneScore groups (Fig. 1F,G). Next, we found that 30 of the 606 DEGs were strongly associated with OS (Table S2). Among the 30 survival-related DEGs, 8 (CXCR2, PTGDR, GPR171, CD3E, CD3D, P2RX1, PNOC and CCL23) were involved in the immune-infiltrating evaluation of CIBERSORT^21^.

### Construction of a prognosis-related risk model based on survival-associated genes

**Figure 2:**
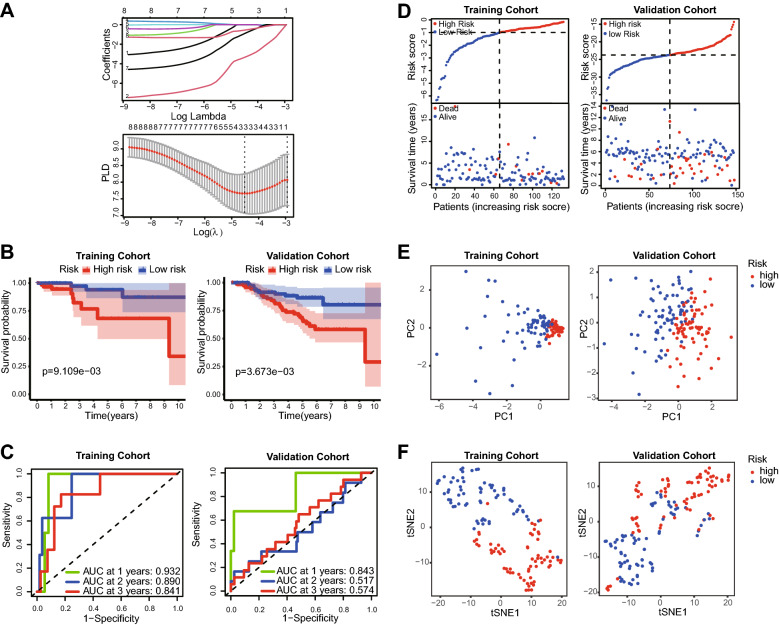
LASSO analysis based on survival-related genes and validation of the established risk model. (**A**) LASSO coefficient profiles (top) of survival-related genes and tenfold cross-validation results (bottom), which identified optimal values of the penalty parameter λ in the training cohort. (**B**) Kaplan–Meier survival curves of patients grouped by the risk score in the training cohort and validation cohort. (**C**) ROC curves of the risk model in training cohort and validation cohort. (**D**) HPBC patients were divided into high- and low-risk score groups according to the risk score model. (**E**,**F**) PCA and tSNE of the risk model in the training cohort and validation cohort.

To precisely evaluate the correlation between immune infiltration and OS, 8 survival-related DEGs were analyzed based on a least absolute shrinkage and selection operator (LASSO) algorithm, and 3 survival-related DEGs (PTGDR, PNOC and CCL23) were selected. Next, the corresponding LASSO coefficients and the optimum value of the penalty parameter *λ* (*λ* = 0.04003709) were acquired (Fig. 2A).

Thus, a prognosis-related risk model was established, and the survival of HPBC patients was assessed according to the risk score calculated based on the expression of the three genes. Risk score = (− 3.372 × PTGDR) + (− 0.948 × PNOC) + (− 0.570 × CCL23). The median risk scores in the different cohorts served as the corresponding cutoff values. Then, all patients were divided into the low- and high-risk score groups. The survival analysis results showed that patients with a low-risk score had a better OS than those with a high-risk scores, with *p* values of 0.009 in the training cohort and 0.0037 in the validation cohort (Fig. 2B). The predictive accuracy of this risk model was assessed by time-dependent receiver operating characteristic (ROC) analysis. The area under the curve (AUC) values were 0.932, 0.890, and 0.841 at 1 year, 2 years, and 3 years, respectively, in the training cohort (Fig. 2C). Similarly, the AUC values were 0.843, 0.517 and 0.574 at the same time points in the validation cohort (Fig. 2C).

All patients were classified based on risk score, OS and living status, as shown in Fig. 2D. Principal component analysis (PCA) and t-distributed stochastic neighbor embedding (t-SNE) analysis were applied to assess the interrelationship between patients in different risk groups (Fig. 2E,F). The results suggested that the established risk model contributed to the accurate classification of HPBC patients.

**Figure 3:**
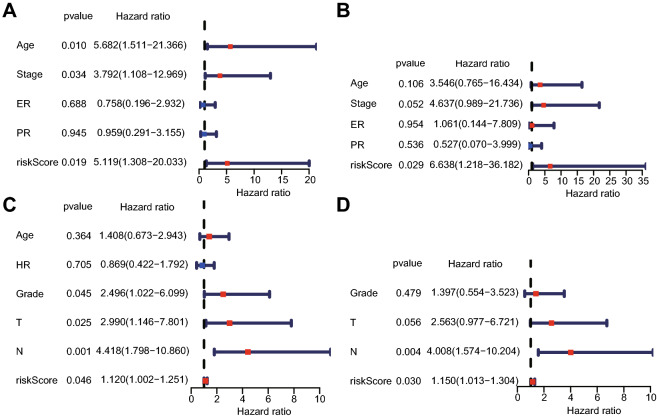
The results of the univariate and multivariate Cox regression analyses. In the training cohort, the forest plots for univariate Cox regression analysis (**A**) show that risk score (high risk vs. low risk), AJCC stage (stages I vs. II, III and IV) and age (< = 50 vs. > 50) were prognostic risk-related variables. The forest plots for multivariate Cox regression analysis (**B**) show that the risk score (high risk vs. low risk) was an independent prognostic factor. In the validation cohort, the forest plots of univariate Cox regression analysis(**C**) and multivariate Cox regression analysis (**D**) indicated that the risk score (high risk vs. low risk) was an independent prognostic factor.

To judge the independent prognostic ability of the risk model, several clinical features, including age, tumor stage (T stage or N stage), histologic grade and ER/PR status, were regarded as binary variables and evaluated by univariate and multivariate analyses. The results suggested a strong association between the risk score and the OS of HPBC patients in the training cohort. The *p* values for the univariate and multivariate analyses were 0.019 and 0.029, respectively (Fig. 3A,B). Similar results were found in the validation cohort; the *p* values for the univariate and multivariate analyses were 0.046 and 0.03, respectively (Fig. 3C,D). These data provided evidence that the risk score can function as an independent prognostic factor to evaluate the OS of patients with HPBC.

### Risk scores correlate with the TIME of HPBC patients

**Figure 4:**
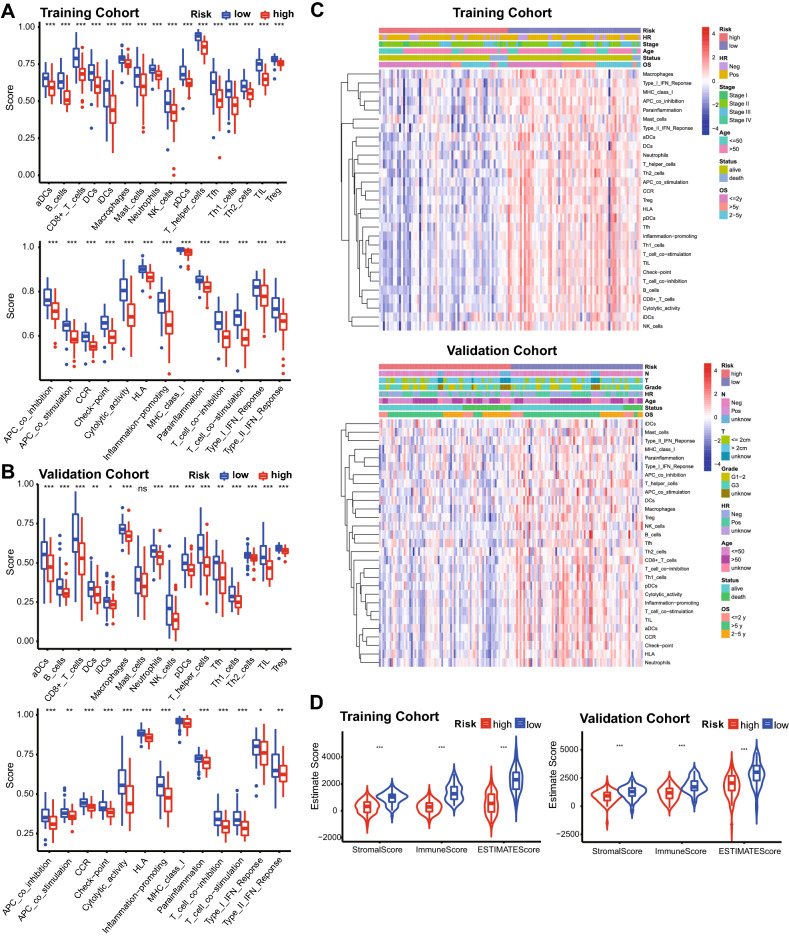
Tumor immune landscape in the high-risk score group and low-risk score group. (**A**) Immune infiltration (top) and immune function (bottom) in the training cohort. (**B**) Immune infiltration (top) and immune function (bottom) in the validation cohort. (**C**) Unsupervised clustering heat map showing the association between immune infiltration and survival-related clinical characteristic parameters, including risk score, age, hormone receptor status, histology grade, tumor stage, OS and survival status, in the training cohort (top) and validation cohort (bottom). (**D**) Comparison of the Estimate scores between the high-risk score group and the low-risk score group in the training cohort and validation cohort.

To investigate the relationship between the risk score and the TIME of HPBC patients, a single-sample gene set enrichment analysis (ssGSEA) algorithm was used to discriminate immune infiltration in HPBC patients. The low-risk score group exhibited more significant immune cell infiltration, including of CD8 T cells, TILs, T helper cells, DCs, aDCs, iDCs, neutrophils, macrophages, NK cells, pDCs and Tregs (Fig. 4A,B). The low-risk score group also demonstrated a more powerful immune response, including checkpoint activity, T-cell coinhibition, T-cell costimulation, APC costimulation, cytolytic activity, CCR, inflammation promotion, parainflammation and APC coinhibition. Based on the ssGSEA score, the CD8 T cell/Treg ratio and CD8 T cell/neutrophil ratio were calculated. As shown in Fig. S3, the CD8 T cell/Treg ratio (*p* < 0.001) and CD8 T cell/neutrophil ratio (*p* < 0.001) were both higher in the low-risk group than in the high-risk group. Furthermore, unsupervised clustering analysis indicated that the patients with a low-risk score almost always harbored greater immune infiltration (Fig. 4C). In addition, the ESTIMATE analysis showed that the low-risk score group possessed a higher StromalScore (*p* < 0.001), ImmuneScore (*p* < 0.001) and ESTIMATEScore (*p* < 0.001) than the high-risk score group in both cohorts (Fig. 4D).

### Identification and functional enrichment of DEGs grouped by risk scores

**Figure 5:**
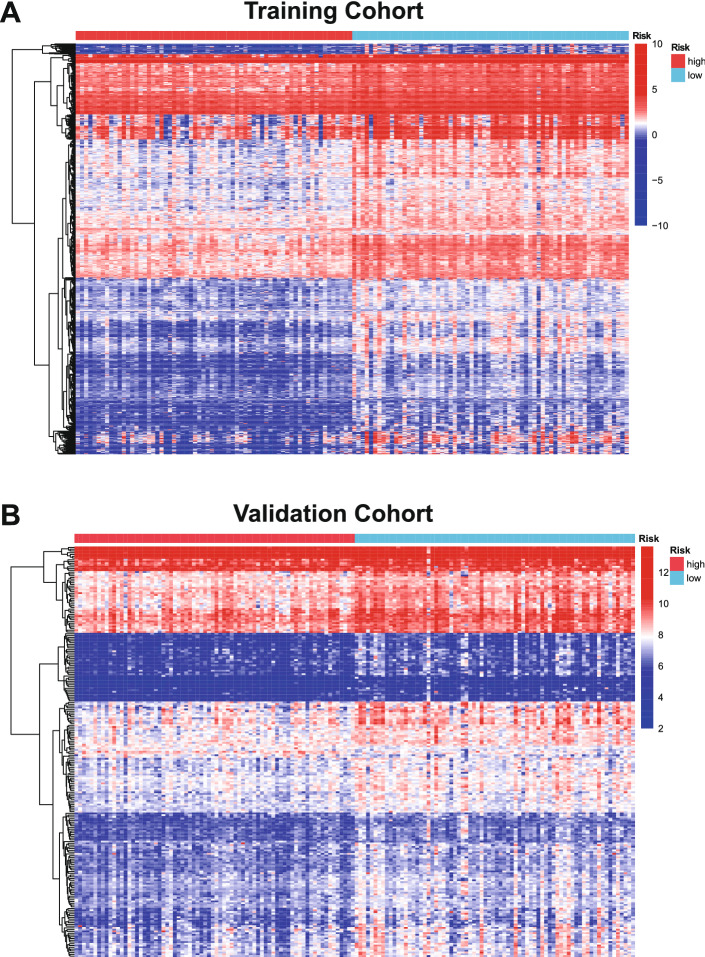
Differentially expressed genes in the training cohort and validation cohort. (**A**) Heatmap of differentially expressed genes between the high-risk score group and the low-risk score group in the training cohort. (**B**) Heatmap of differentially expressed genes between the high-risk score group and the low-risk score group in the validation cohort.

Next, we analyzed the DEGs between different risk score groups in both cohorts. Our results showed that 3338 DEGs were identified in the training cohort (*p* < 0.05, Fig. 5A), and 1205 DEGs were identified in the validation cohort (*p* < 0.05, Fig. 5B). Among these genes, 322 overlapping DEGs were found with the cutoff *p* < 0.01 (Fig. S2).

**Figure 6:**
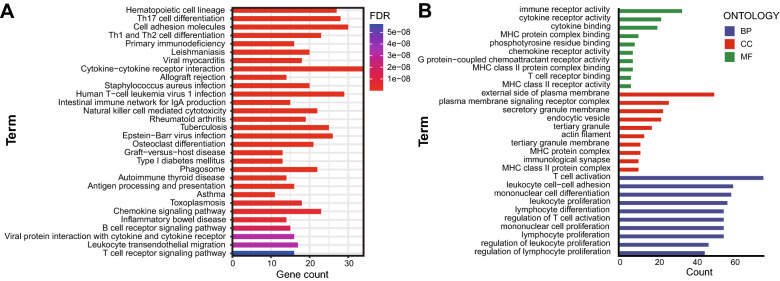
KEGG and GO analysis of differentially expressed genes co-existing in the training cohort and validation cohort. (**A**) Representative results of KEGG pathway analysis of differentially expressed genes. (**B**) Representative results of GO enrichment analysis of differentially expressed genes.

**Figure 7:**
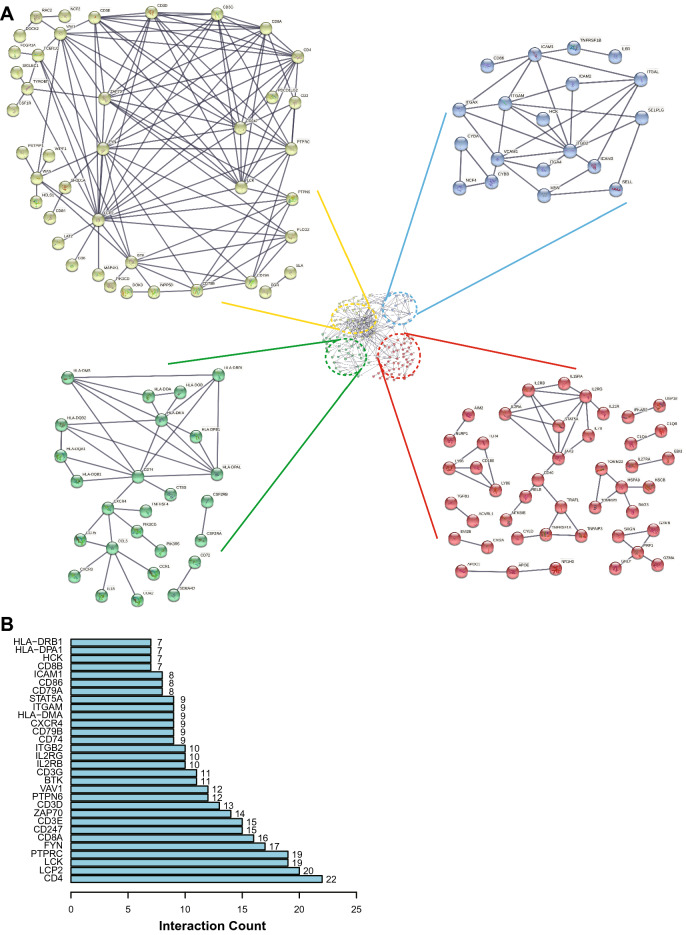
The results of protein–protein interactions of differentially expressed genes co-existing in the training cohort and validation cohort. A total of 322 differentially expressed genes were analyzed in the STRING database, a network (**A**) of PPIs was acquired (the confidence was 0.95), the different colors represented different subgroups after PPI analysis, and the top 30 genes (**B**) were ranked by interaction count.

Furthermore, the potential functions of 322 DEGs were evaluated according to Kyoto Encyclopedia of Genes and Genomes (KEGG) and gene ontology (GO) enrichment analyses. KEGG analysis showed that immune-related pathways (including cytokine–cytokine receptor interaction pathway, antigen processing and presentation, B cell receptor signaling pathway and chemokine signaling pathway) are potentially the most important mechanisms. (Fig. 6A). GO analysis suggested that these overlapping DEGs primarily mediated immune-related biological functions (Fig. 6B). These data showed that the important biological processes included T cell activation, lymphocyte proliferation, regulation of T cell activation, lymphocyte differentiation and positive regulation of lymphocyte activation. In addition, the enriched cellular component and molecular function categories are in Fig. 6B, these included external side of plasma membrane, plasma membrane signaling receptor complex, immunological synapse, immune receptor activity, cytokine receptor activity and MHC protein complex binding. Furthermore, GSEA was performed to map all DEGs. As shown in Fig. S4, in both the training and validation cohorts, immune-related pathways and biological functions were the crucial factors, and were enriched in the low-risk group. To observe interactions among these 322 overlapping DEGs, the STRING protein–protein interaction (PPI) database was used with an interaction score greater than 0.95. As shown in Fig. 7A, all the DEGs were divided into four groups: immune cell migration (green), immune cell activation and maturation (red), immune cell binding (blue) and intracellular signal transduction (yellow). The top 30 genes ranked by interaction count are shown in Fig. 7B; these genes included CD4, LCP2, LCK, PTPRC and FYN. Taken together, the evidence indicates that these DEGs based on the risk model may play a critical role in the tumor immune profile in HPBC patients.

**Figure 8:**
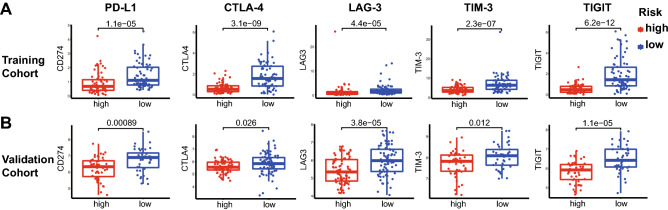
Expression of immune checkpoint genes in different risk groups. (**A**) The expression of immune checkpoint genes, including PD-L1, CTLA-4, LAG-3, TIM-3 and TIGIT, in the training cohort. (**B**) The expression of the same immune checkpoint genes in the validation cohort.

Finally, we evaluated the importance of immune checkpoint-related genes, such as PD-L1, LAG-3, CTLA-4, TIM-3 and TIGIT. The results demonstrated that the expression of these 5 genes was significantly upregulated in the low-risk score group of the training cohort (all *p* values < 0.001, Fig. 8A). As expected, similar differences were found in the validation cohort (*p* < 0.001, *p* = 0.026, *p* < 0.001, *p* = 0.012, and *p* < 0.001, respectively; Fig. 8B).

## Discussion

At present, tumor immunotherapy is an important treatment option, that is widely utilized to treat various malignancies. However, this revolutionary therapeutic regimen has not achieved great breakthroughs in patients with HPBC. Therefore, it is crucial to distinguish the tumor immune-infiltrating profile in HPBC patients, and in the future, we will likely provide immunotherapy for appropriate patients according to TIME analysis. In our study, we initially utilized the ESTIMATE algorithm to assess tumor immune infiltration in patients with HPBC. Our results indicated a strong association between tumor immune infiltration and the prognosis of HPBC patients based on the ImmuneScore. Unfortunately, the ImmuneScore could not serve as an independent marker with predictive prognostic ability.

In recent years, risk models with the ability to predict prognosis have gained increasing attention. Many risk models are established based on gene expression profiles and are widely utilized in studies of various malignancies, such as breast cancer^22^ and lung adenocarcinoma^23^. Hence, after 8 differentially expressed genes based on ESTIMATE scores were verified to be significantly associated with survival and the immune-infiltrating assessment of CIBERSORT, the LASSO algorithm was employed to select optimal genes. Finally, 3 genes including PTGDR (prostaglandin D2 receptor), PNOC (prepronociceptin) and CCL23 (C–C motif chemokine ligand 23), were selected as core entities of this model. These genes are classified as immune-related genes in the Immunology Database and Analysis Portal (Imm-Port) (https://immport.niaid.nih.gov)^24^. PTGDR, known as the receptor of prostaglandin D2, contains 2 subtypes: prostaglandin D2 receptor 1 (DP1) and prostaglandin D2 receptor 2 (DP2). PTGDR may serve as an immune inhibition-related gene. A few studies have shown that PTGDR might suppress the function of natural killer cells^25^ and promote an increase in Foxp3^+^ CD4^+^ regulatory T cells^26^. Accumulating data suggest that hypermethylation of PTGDR might play a role in the tumorigenesis and development of various cancers, including bladder cancer^27^, colon cancer^28^, lung adenocarcinoma^29^, cervical cancer^30^, endometrial carcinoma^31^ and gastric cancer^32^. PNOC encodes a preproprotein that is proteolytically processed to generate multiple protein products, including nociceptin, nocistatin, and orphanin FQ2 (OFQ2)^33^. It has been reported that nociception/orphanin FQ2 (N/OFQ) might be activated by the proinflammatory cytokines, interleukin-1 beta (IL-1beta) and tumor necrosis factor-alpha (TNF-alpha) via the ERK 1/2 and p38 MAP kinases pathways^34^. Further, N/OFQ interacts with nociceptin receptor (NOP) in cancer, and promotes the proliferation and invasion of tumor cells via the PI3K/Akt signaling pathway^35^, it also functions as an immune inhibitor by binding the N/OFQ peptide receptor on the surface of circulating immune cells^36^ or inhibiting the activation of DCs^37^. Published clinical data showed that NOP overexpression implied a poor prognosis in non-small-cell lung cancer^35^. However, until now, few studies have verified the direct association between PNOC and cancer. Thus, we hypothesize that PNOC may indirectly mediate immune inhibition and promote tumor growth by N/OFQ. CCL23 is a member of the CC chemokine family and demonstrates chemotactic activity on resting T lymphocytes, monocytes, neutrophils^38^ and dendritic cells^39^ but is not expressed on activated T lymphocytes. In addition, human neutrophils orchestrate the recruitment of different cell types to inflamed sites by releasing CCL23, which then controls the immune response^40^. There is much evidence that CCL23 expression is significantly upregulated in various cancers, including lung cancer^41^, colorectal cancer^42,43^, and ovarian cancer^44^. CCL23 could promote ovarian cancer migration by activating the ERK1/2 and PI3K pathways^44^. Furthermore, CCL23 expression exhibited positive correlation with PD-L1 expression^45^. Hence, we hypothesize that CCL23 chiefly acts as an immune inhibitor and promotes tumor growth. However, a liver cancer study showed that CCL23 could suppress tumor progression by promoting the immune infiltration of CD8 T cells^46^.

According to the median risk score, all patients were classified into the high- and low-risk score groups. The predictive capacity of the risk model was verified to be precise and powerful. The patients in different cohorts were grouped by the respective median risk score, because there was a great difference in the range of the risk scores between the two cohorts. This difference in median risk score might be caused by detection platform, detection batch, and geographic area.

The ssGSEA algorithm was broadly employed to assess the TIME. A higher ssGSEA score indicated a better immune-infiltrating profile and potentially a better prognosis. In our study, the low-risk score group demonstrated strong immune infiltration, including of CD8 T cells, TILs, T helper cells, DCs, aDCs, iDCs, neutrophils, macrophages, NK cells, pDCs and Tregs. Numerous studies have confirmed that many subtypes of immune cells contribute to promoting the antitumor immune response. DCs play a crucial role during T-cell priming by presenting processed MHC antigenic peptides^47^. In addition, macrophages and NK cells act as part-time antigen presenting cells and contribute to T-cell priming^48^. CD8 T cells and TILs professionally execute cytotoxic effects on tumor cells, and T helper cells were confirmed to kill tumor cells via antigen cross-presentation^49^. Hence, we confirmed the presence of a better tumor-infiltration profile in the TIME of patients with a low-risk score.

Unexpectedly, the proportions of neutrophils and Tregs, which are regarded as immunosuppressive cells, were higher in the patients with a low-risk score. Most studies provide evidence that an increased neutrophil or Treg frequency would imply poor outcome or survival. Nonetheless, contrasting evidence has been published. Some research found that high infiltration of Tregs could improve overall survival in Hodgkin's lymphoma^50,51^, follicular lymphoma^52^, head and neck cancer^53^ and breast cancer^54^. Several authors proposed that Tregs are not a homogenous population, and can be divided into several subgroups that differ in function according to surface antigen expression^55^. Therefore, Tregs appear to play dual roles in cancer and cannot accurately predict prognosis^56^. Recently, the TIL/Treg ratio and TIL/neutrophil ratio were reported to be powerful prognostic indicators of survival or treatment outcome in breast cancer^57,58^ and other cancers^59,60^. In our study, the low-risk group had a higher CD8 T cell/Treg ratio and CD8 T cell/neutrophil ratio. This result suggested that the low-risk group might have a better prognosis and was consistent with the survival analysis results.

As expected, the low-risk score group showed signs of a more powerful immune response, including checkpoint activity, T-cell coinhibition, T-cell costimulation, APC costimulation, cytolytic activity, CCR, increased inflammation, parainflammation and APC coinhibition. Publication of the theory of the T-cell cosignaling pathway was an important milestone in the field of immunology^61^, and this pathway includes both coinhibitory and costimulatory signaling pathways^62^. Accumulating evidence has proven that cosignaling molecules (either coinhibitory receptors or costimulatory receptors) direct T-cell function and determine T-cell fate after T cells are activated by the TCR signaling pathway^62^. In essence, cosignaling molecules are immune checkpoints. Previous studies have reported that enhanced cytolytic activity is a feature of ADCC mediated by activated T cells^63^ or NK cells^64^. Activated T cells migrate directly into tumor tissue along the chemokine gradient and kill tumor cells. Chemokine receptors (CCRs) expressed on the surface of T cells mediate this migration of T cells by binding the corresponding chemokine^65^. Overall, the immune function profile in HPBC patients with a low-risk score might suggest an increased antitumor immune response.

Tumor tissue composition including of stromal cells and immune cells, is strongly correlated with the OS of cancer patients^66^. Coincidentally, the ESTIMATE algorithm could assess the overall constitution of tumor tissue, including stromal cells and immune cells. Furthermore, several studies have validated that the ESTIMATE score might accurately map the TIME and precisely predict the OS of patients with cancer^67^. Similar to the ssGSEA results, our ESTIMATE analysis results support that patients with a low-risk score harbored a better immune-infiltration profile.

Subsequently, we tried to reveal the potential mechanism that promotes antitumor immune infiltration and improves the OS of patients with HPBC. A total of 322 DEGs overlapped between the two cohorts. We thought that these genes might play crucial roles during tumorigenesis and progression. Further KEGG analysis indicated that these DEGs were involved in immune-related pathways. Meanwhile, the top GO terms suggested that these genes might mediate the entire T-cell immune response, including priming, activation, proliferation, migration, recognition and killing. In addition, the gene–gene interaction analysis results strongly supported the importance of immune-related signaling pathways. Moreover, GSEA revealed an overview of the TIME in HBPC patients. We further verified the difference in the TIME between the low- and high-risk groups and hypothesized that this difference contributes to survival in the low-risk score group.

At present, immune checkpoints including PD-L1, CTLA-4, LAG3, TIGIT and TIM-3, are thought to contribute to tumor immune escape. High expression of these immune checkpoints indicates poor prognosis; however, some different opinions have been published recently. Shang et al.^68^ reported that HPBC patients with high PD-L1 expression of tumor cells had a better prognosis after neoadjuvant chemotherapy. Ni et al.^69^ found that PD-L1 expression of TILs implied a good prognosis in breast cancer patients. Lee et al.^70^ reported that high LAG-3 mRNA levels were associated with high levels of TILs in HPBC. High infiltration of TILs is a marker of favorable prognosis. In this study, PD-L1, LAG3, TIGIT, TIM-3 and CTLA-4 were upregulated in the low-risk group. We speculated that high expression of these immune checkpoints might be associated with TIL-mediated antitumor inflammatory responses^71^. However, our study indicated that multiple coinhibited signaling pathways might be involved in the tumorigenesis of HPBC patients with a low-risk score, and combination treatment with multiple immune checkpoint inhibitors may achieve better outcomes in these patients.

In our study, we established and validated a prognosis-related risk model composed of three survival-related genes that were strongly associated with immune infiltration. More importantly, we found that immune escape caused by multiple immune cosignaling pathways might underlie oncogenesis and tumor progression in some patients with HPBC. Hence, we speculated that combination regimens of multiple immune checkpoint inhibitors might achieve breakthroughs in the treatment of HPBC patients in the future. However, there were some limitations of our study. First, this study lacked validation with experimental data. Moreover, some analytical biases were inevitable due to the absence of some clinical data. Therefore, an analysis of clinical samples was necessary to consolidate the conclusions of this study.

## Methods

### Data acquisition and preprocessing

We obtained all data (including clinical characteristics and mRNA expression) of HPBC patients from the TCGA database (https://portal.gdc.cancer.gov/) and GEO database (https://www.ncbi.nlm.nih.gov/geo/). The TCGA dataset was considered the training cohort, and the GSE20711, GSE45255, GSE162228 and GSE1456 GEO datasets were regarded as the validation cohorts. Patients with an OS of less than 1 month were excluded from the analysis. Thus, 132 HPBC patients in the TCGA and 148 HPBC patients in GEO were accepted for subsequent study. The procedures involving with the data collection and analyses abided by all provisions of the TCGA and GEO.

### Assessment of the TIME in HPBC patients

The ESTIMATE algorithm is appropriate for assessing the profile of the TIME, which contains stromal cells and immune cells^20^. A higher score indicates more significant infiltration of the corresponding component in the tumor tissue. The ssGSEA algorithm was applied to judge the level of immune cell infiltration and the immune response mechanism based on published data from Bindea et al.^72^. Thus, these analyses provided an overview of the tumor-infiltrating landscape.

### Identification of DEGs and the analysis of potential functions

The DEGs between different groups were mapped by the “limma” R package. Genes with a *p* value < 0.05 were regarded as DEGs.

Functional GO and KEGG analyses^73^ of the DEGs were performed utilizing the “clusterProfiler” package. Then, the potential mechanisms were elucidated according to FDR < 0.05. All the data were visualized by the “ggplot2” and “topGO” packages.

We carried out gene set enrichment analysis (GSEA) using GSEA software (V4.1.0) to determine whether DEGs exhibited significant and consistent differences between the high- and low-risk groups^74^.

### Establishment of a risk model with prognostic ability

The optimal survival-related genes in the training cohort were selected by LASSO analysis based on the “glmnet” R package^75^. Tenfold cross-validation was used for filtering, and the λ value was obtained. Meanwhile, the coefficients of the selected genes were acquired. Then, the risk score of each sample was regarded as the summation of the product of each selected gene and the corresponding coefficient. Thus, all patients were classified into the high-risk score or low-risk score group based on the respective median risk scores in both cohorts.

Subsequently, the survival difference between the two groups was analyzed and plotted by the “survival” and “survminer” R packages. The “survival ROC” R package was applied to determine the AUC value and plot the ROC curve. PCA and t-SNE were utilized to analyze the clustering of each sample in the model. Furthermore, the risk score was regarded as a prognosis-related variable, and the independent predictive power of this variable was assessed by univariate and multivariate analyses using the “survival” package.

### Statistical analysis

All statistical analyses were performed using R software (version 4.1.1) (https://www.r-project.org/). The differences between variables in two groups were examined by using Wilcoxon’s test. Kaplan–Meier curves were utilized to assess the survival data. Independent prognostic factors were judged via univariate and multivariate Cox regression analyses. *p* < 0.05 was considered to indicate statistical significance (*p < 0.05; **p < 0.01; ***p < 0.001).

### Ethics approval and consent to participate

Not applicable. All data in this study are publicly available.

## Supplementary Information

Supplementary Information.

## Data Availability

All data obtained for this study can be found in the TCGA (https://portal.gdc.cancer.gov/) and Gene Expression Omnibus (GEO) repository (https://www.ncbi.nlm.nih.gov/geo/).
