# Supplementary material for: Evaluating the tumor immune profile based on a three-gene prognostic risk model in HER2 positive breast cancer
Source: Sci Rep. 2022 Jun 3;12:9311. doi: 10.1038/s41598-022-13499-1 (PMC9166798; doi:10.1038/s41598-022-13499-1)
Supplement: Supplementary file 1 — Supplementary Information. [file 41598_2022_13499_MOESM1_ESM.pdf]

Evaluating The Tumor Immune Profile Based on A Three-Gene Prognostic Risk Model in HER2 Positive Breast Cancer

Jianqing Lin [1], Aiyue zhao [2], Deqiang Fu [2*]

[1] Thyroid & Breast Surgery, The Second Affiliated Hospital of Fujian Medical University, Quanzhou, China.

[2] Department of Oncology, The Second Affiliated Hospital of Fujian Medical University, Quanzhou, China.

[*] Correspondence and requests for materials should be addressed to DQF (Email: honglai1999@fjmu.edu.cn)

Supplementary material

Figure S1

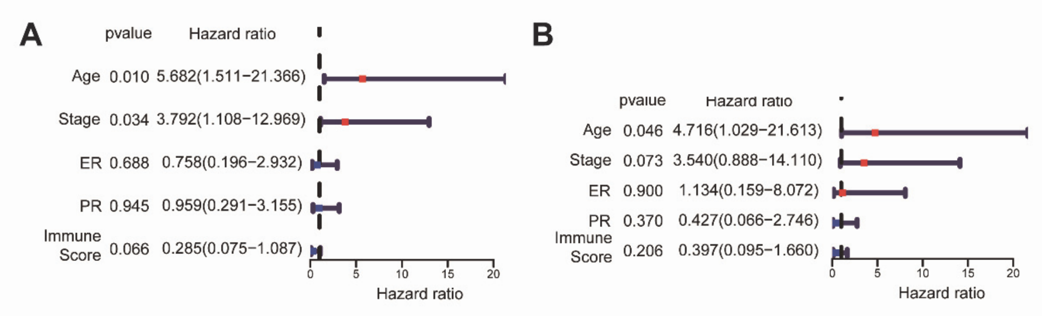

Figure S1 The results of univariate and multivariable Cox regression analysis in training cohort. Univariate Cox regression (A) was applied to assess the predictive power of prognosis-related parameters including age (<=50 vs. >50), AJCC stage (stage I vs. II, III and IV), ER status (positive vs. negative), PR status (positive vs. negative) and ImmuneScore (high vs. low). Multivariable Cox regression (B) was utilized to evaluate the independence of these parameters.

Figure S2

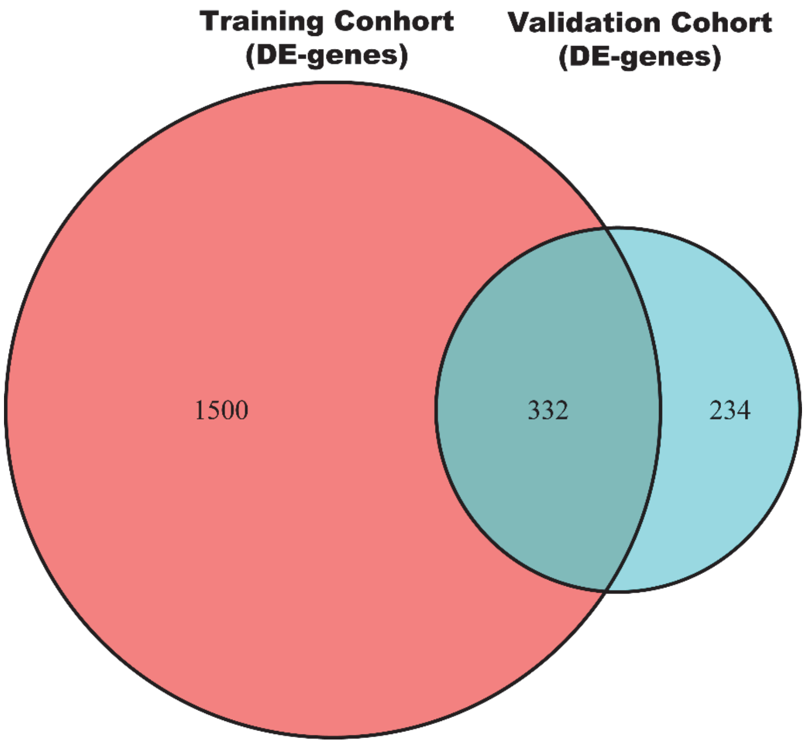

Figure S2 Venn diagram of the differentially expressed genes (*p*<0.01) between different risk groups from training cohort and validation cohort.

Figure S3

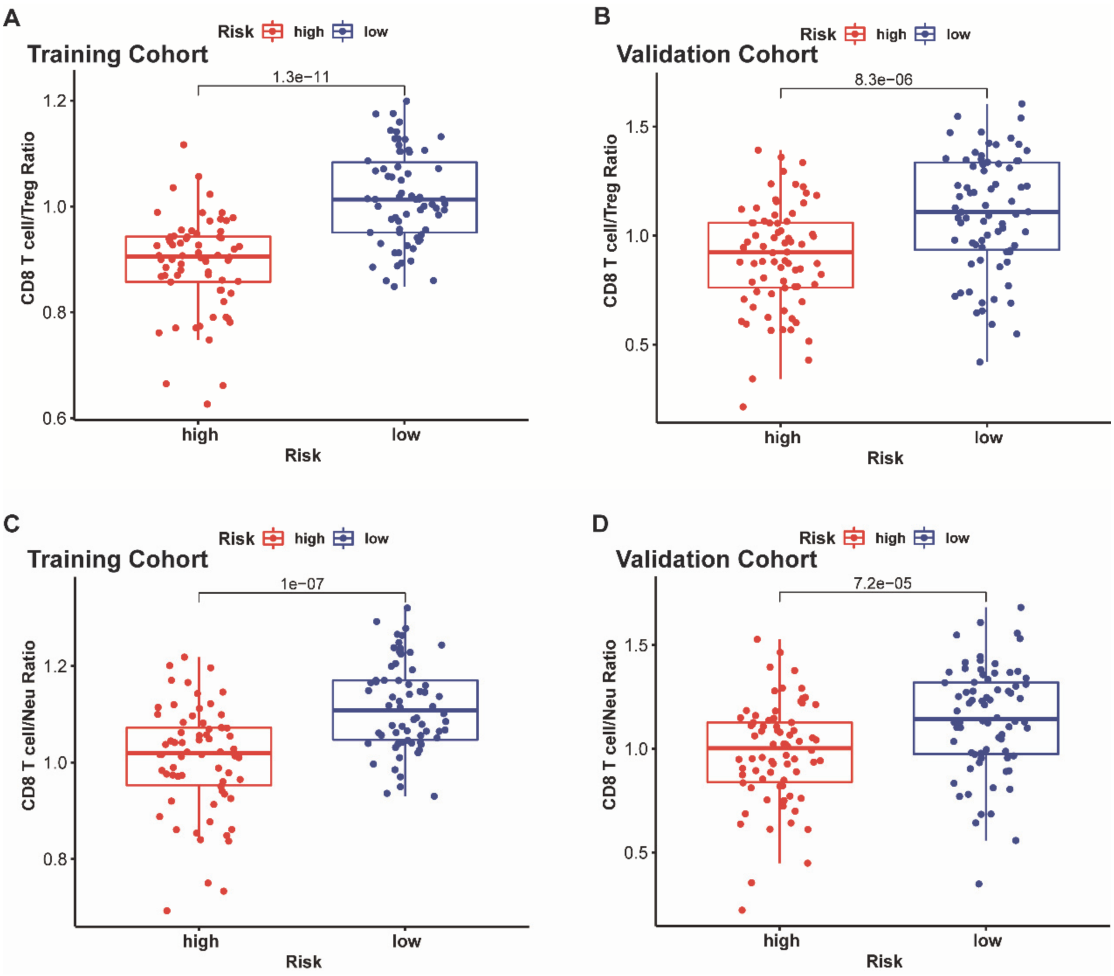

Figure S3 CD8 T cell/Treg ratio and CD8 T cell/Neu Ratio in training cohort and validation cohort.

Figure S4

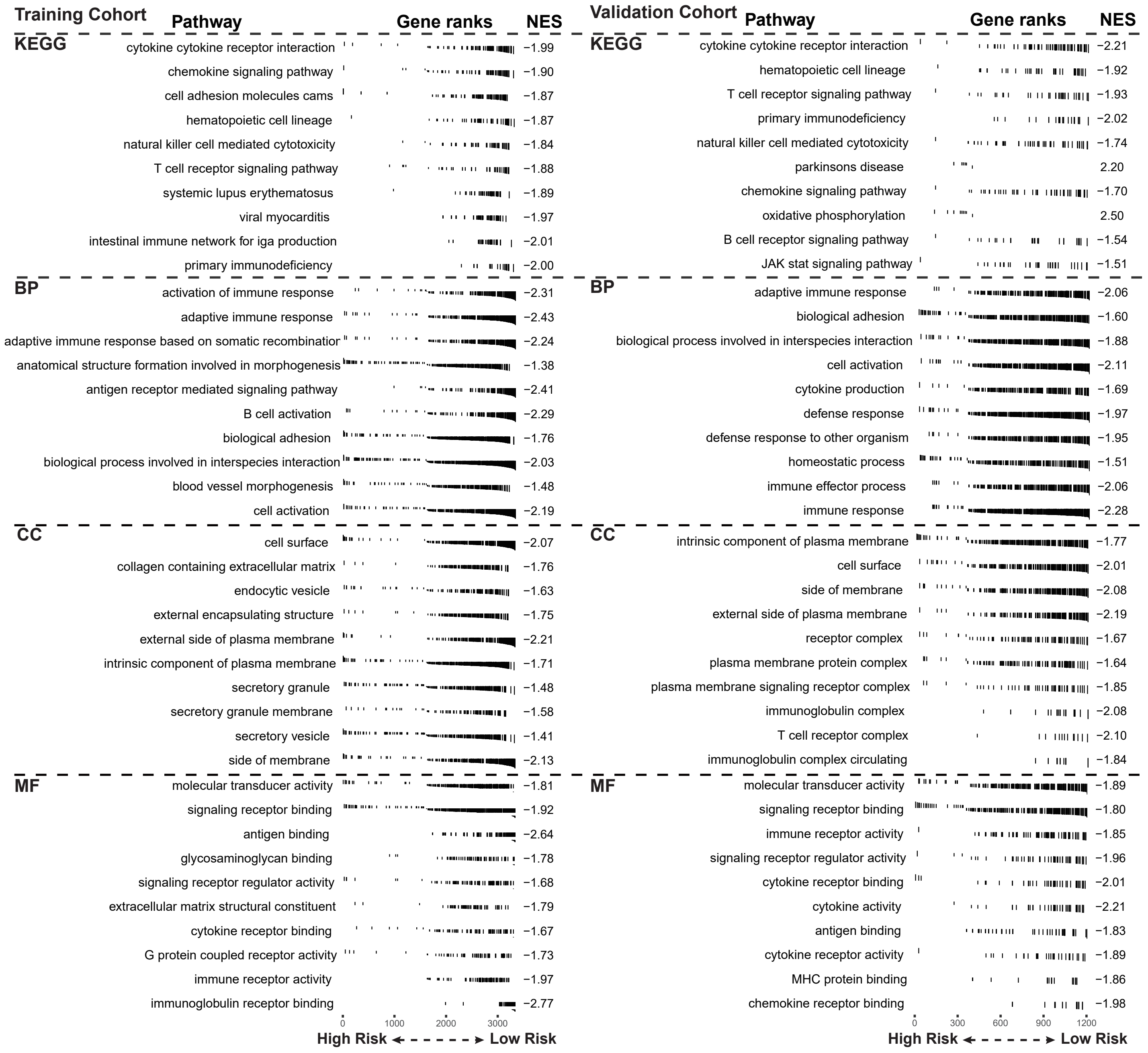

Figure S4 GSEA analysis of DEGs (p<0.05) in training cohort and validation cohort.

Table S1 Clinicopathological characteristics of patients with HPBC in training cohort and validation cohort.

Clinical Features	Training cohort	Validation cohort			
	TCGA	GSE20711	GSE45255	GSE162228	GSE1456
	(n = 132)	(n = 26)	(n = 61)	(n = 46)	(n = 15)
**Age (years)**					
<= 50	40 (30.3%)	10 (38.5%)	24 (39.3%)	21 (45.7%)	unknown
> 50	92 (69.7%)	16 (61.5%)	37 (60.7%)	25 (54.3%)	unknown
median	60.5	unknow	54	52	
**Histological Grade**					
G1	0	0	2 (3.3%)	0	unknown
G2	0	2 (7.7%)	22 (36.1%)	21 (45.7%)	unknown
G3	0	24 (92.3%)	36 (59%)	25 (54.3%)	unknown
unknow	132 (100%)		1 (1.6%)		unknown
**T Stage**					
T1	28 (21.2%)	8 (30.8%)	13 (21.3%)	unknown	unknown
T2-4	104 (78.8%)	18 (69.2%)	48 (78.7%)	unknown	unknown
**N Stage**					
N negative	45 (34.1%)	4 (15.4%)	36 (59%)	unknown	unknown
N positive	87 (65.9%)	22 (84.6%)	25 (41%)	unknown	unknown
**Stage**					
**0**		unknown	unknown	4 (8.7%)	unknown
I	13 (9.8%)	unknown	unknown	10 (21.7%)	unknown
II	80 (60.6%)	unknown	unknown	18 (39.1%)	unknown
III	35 (26.5%)	unknown	unknown	13 (28.3%)	unknown
IV	2 (1.5%)	unknown	unknown	1 (2.2%)	unknown
unknow	2 (1.5%)				
**HR Status**					
HR negative	33 (25%)	17 (65.4%)	23 (37.7%)	26 (56.5%)	unknown
HR postive	99 (75%)	8 (30.8)	38 (62.3%)	20 (43.5%)	unknown
unknow		1 (3.8%)			
**Survival (years)**					
<= 2	68 (51.5%)	5 (19.2%)	11 (18%)	7 (15.2%)	1 (6.7%)
2-5	39 (29.6%)	4 (15.4%)	22 (36.1%)	11 (23.9%)	6 (40%)
> 5	25 (18.9%)	17 (65.4%)	28 (45.9%)	28 (60.9%)	8 (53.3%)
median	1.861	6.005	4.83	5.4	
**Survival status**					
Alive	119 (90.2%)	15 (57.7%)	50 (82%)	37 (80.4%)	8 (53.3%)
Dead	13 (9.8%)	11 (42.3%)	11 (18%)	9 (19.6%)	7 (46.7%)
**Anti-Her2 therapy**					
yes	58 (43.9%)	unknown	unknown	unknown	unknown
no	74 (56.1%)	unknown	unknown	unknown	unknown

**Chemotherapy**					
yes	79 (59.8%)	unknown	32 (52.5%)	unknown	unknown
no	53 (40.2%)	unknown	29 (47.5%)	unknown	unknown
**Endocrinotherapy**					
yes	63 (47.7%)	unknown	33 (54.1%)	unknown	unknown
no	69 (52.3%)	unknown	28 (45.9%)	unknown	unknown

Table S2 The survival-related differentially expressed genes from training cohort.

Gene	ENTREZID	P value
GPR150	285601	0.027
JCHAIN	3512	0.032
PYHIN1	149628	0.042
SCGB3A2	117156	0.046
P2RX1	5023	0.027
CD53	963	0.048
TRAV23DV6	28660	0.042
TRG-AS1	100506776	0.039
IGHV3-47	28425	0.045
CXCR2	3579	0.011
IGHG3	3502	0.046
SLA	6503	0.019
PTGDR	5729	0.016
IGKV1OR22-1	3530	0.027
PARP15	165631	0.046
CCL23	6368	0.047
DPT	1805	0.041
IGHV1-12	28471	0.02
CLLU1OS	574016	0.046
PRKCQ	5588	0.049
CD3E	916	0.019
XPNPEP2	7512	0.047
IGHJ3	28479	0.025
CD3D	915	0.02
TRAV8-3	28683	0.047
TRAV39	28642	0.045
PNOC	5368	0.028
ADGRE4P	326342	0.021
GPR171	29909	0.017
TRAV19	28664	0.04
